# 

**Published:** 1888-10

**Authors:** 


### CONTENTS—OCTOBER, 1888.—VOL. IV., NO. 4.

ORIGINAL ARTICLES— Page.

Pathology and Treatment of Chronic Cervical Endometritis. By J. W. McLaughlin, M. D., Austin . . . . . 139

Address of the retiring President A. D. M. S., Thomas Wooten, M. D., Austin . . . . . . . . . . . . . . . 151

Placenta Prævia, a case complicated with Twins. By Sam Cunningham, M. D., Elgin . . . . . . . . . . 158

SOCIETY NOTES—

Physicians' Mutual Benefit Association of Texas; Mrs. Harrington's benefit, receipt, and list of "paid." . . . 164

Austin District Medical Society; Annual Meeting and Banquet, etc. . . . . . . . . . . . . . . . . . . . . 165

American Association of Obstetricians and Gynecologists. 166

East Line Medical Society of Texas . . . . . . . . . 166

After the Quacks in Van Zandt . . . . . . . . . . . . 166

CULLINGS FROM CONTEMPORARIES—

Edited by Dr. T. J. Bennett, Austin. A variety of practical points. . . . . . . . . . . . . . . . . . . . . 167

EDITORIAL—

"It Smells to Heaven." Hog's Lard vs. Cotton Seed Oil. 169

The Treatment of Yellow Fever . . . . . . . . . . . 171

Death of Dr. C. K. Gregg . . . . . . . . . . . . . . 178

Death of Dr. Early W. Walton . . . . . . . . . . . . 179

A Sensation in Medical Circles . . . . . . . . . . . 179

MEDICAL NEWS AND MISCELLANY—

A large number of interesting items . . . . . . . . . 175

PUBLISHERS NOTES—

Editorial and other mention of certain firms, preparations, institutions, etc.,—very interesting and important to be read. . . . . . . . . . . . . . . . . . . . . . . . 181

ERRATA . . . . . . . . . . . . . . . . . . . . . . 176

